# Optimization of Washing Processes in Solvothermal Synthesis of Nickel-Based MOF-74

**DOI:** 10.3390/ma13122741

**Published:** 2020-06-17

**Authors:** Khaliesah Kamal, Mohamad Azmi Bustam, Marhaina Ismail, Denys Grekov, Azmi Mohd Shariff, Pascaline Pré

**Affiliations:** 1 CO_2_ Research Centre, CO2RES, Department of Chemical Engineering, Universiti Teknologi PETRONAS, Perak 32610, Malaysia; nurulkhaliesah@imt-atlantique.fr (K.K.); marhaina_19001049@utp.edu.my (M.I.); azmish@utp.edu.my (A.M.S.); 2 GEPEA Laboratory, Department of Environment and Energy Systems, IMT Atlantique, UMR-CNRS 6144, 44300 Nantes, France; denys.grekov@imt-atlantique.fr; 3 Centre of Research in Ionic Liquids, CORIL, Department of Chemical Engineering, Universiti Teknologi PETRONAS, Perak 32610, Malaysia

**Keywords:** metal-organic frameworks, Ni-MOF-74, solvothermal synthesis, product washing, pore activation, centrifugal separation

## Abstract

Solvothermal synthesis is the most preferable preparation technique of metal–organic frameworks (MOFs) that consists of reactants mixing, ultrasonication, solvothermal reaction, product washing, and solvent evacuation. Owing to fast reaction kinetics in solvothermal reaction, this technique allows for production of uniform MOF particles with high crystallinity, high phase purity, and small particle sizes. However, it exhibits some difficulties of washing processes that may involve the blockage of pores due to incomplete removal of reactive medium from MOF products. The present study proposes an improvement of washing processes by introducing centrifugal separations with optimized parameters at two different stages: after reaction and after product washing. Nickel-based MOF-74 was synthesized as the experimental material for this purpose. The quality of the produced sample was evaluated by gas adsorption performance using CO_2_ at 1 bar and 25 °C. The final sample of the optimized synthesis routes was able to adsorb 5.80 mmol/g of CO_2_ uptake, which was competitive with literature data and significantly higher than the sample of the basic synthesis. Fourier-transform infrared spectroscopy (FTIR) and powder X-ray diffraction (PXRD) analysis revealed that the sample displayed much higher crystallinity structure and was clean from impurities after centrifugations. The outcome indicated the success of separation between MOF products and reactive medium during washing processes, leading to the effective pore activation of MOFs.

## 1. Introduction

Metal-organic frameworks (MOFs) are the assembly of metal-containing nodes coordinated to organic bridging ligands giving rise to characteristic three-dimensionally ordered crystalline structures [1]. In recent years, the research area of these materials has grown tremendously, leaving behind other porous solids due to its superior potential advantages. MOFs are recognized for their ability to provide large nanoporous volumes and immense specific surface areas for numerous applications [2]. MOF pore structures are able to act as catalytic sites allowing their application in nanocatalysis [3]. MOF magnetic properties and their different adsorption behavior towards various gases allow their application in gas sensing [4]. The excellent biocompatibility of MOFs and their ability to afford high amounts of drug loading allow their application in drug delivery [5]. MOFs also can be employed for removal of hazardous substances [6] and key odorants [7], development of electrode materials for electrochemical detectors and energy storage devices [8], and as luminescent materials in the application of lighting, organic pigments, as drug tracers, and many others [9]. Furthermore, one of the most promising applications of MOFs is gas storage and separation relying on adsorption [10]. For instance, the Ni-MOF-74 family appears to be among one of the best adsorbent materials for CO_2_ adsorption originated from strong intermolecular interactions with exposed metallic centers in addition to their nanoporous volumes [11,12,13].

There are six common preparation routes of MOFs that can be classified: solvothermal synthesis, microwave assisted synthesis, sonochemical synthesis, mechanochemical synthesis, electrochemical synthesis, and, slow evaporation synthesis [10,13,14,15,16,17]. Solvothermal synthesis is the most preferable method being used in approximately 70% of published works on MOF materials [10,17]. In general, solvothermal preparation of MOFs consists of employing soluble metal salts, organic linkers, and organic high-boiling solvent in a sealed vessel and heated above the boiling point of the solvent to undergo the reaction. Then, the product is recovered and washed, followed by the evacuation of solvent in MOF pores. Owing to the fast reaction kinetics in solvothermal reaction, this technique allows production of uniform MOF particles with high crystallinity, high phase purity, and small crystallite size distribution, requiring however thorough optimization of experimental conditions [17,18]. One should notice that for different applications of MOF materials involving the processes of mass transfer, aside from highly specific surface area and microporous volume, average particle size and size distribution are important parameters determining the kinetics of the process and diffusion barriers. In the particular case of gas adsorption, the diffusion barrier of mass transfer decreases with crystallite sizes [19].

However, solvothermal synthesis undeniably exhibits some shortcomings, such as the use of expensive stainless steel autoclaves and Teflon reactors, environmental hazards with the use of solvents, limitation of mass production per synthesis, and difficulties of product washing processes [18]. The latter involves the use of solvents such as dimethylformamide, diethylformamide, acetonitrile, acetone, ethanol, and methanol, where the mixtures of solvents have also been used in many cases to avoid problems of different solubility for the different reactants [10]. The chemical reaction takes place between inorganic and organic components leading to the formation of frameworks of the material contoured by the solvent molecules that act as a “pore template”, and will be removed during washing and evacuation processes at high temperatures [20]. Depending on the operation conditions, the processes may involve the blockage of pores due to the incomplete removal of reactive medium from MOF product such as the solvents of reaction, residual reactants, and side products [20].

For that reason, the present study proposes an optimization of washing techniques in solvothermal synthesis of MOFs. Nickel-based MOF-74 (Ni-MOF-74) was synthesized as the experimental material for this purpose. The efficiency of pore activation in the synthesis resulted from the quality of separation between MOF products and reactive medium in washing processes. It was then evaluated by the performance of the produced sample for CO_2_ adsorption, which was the interested application of this material.

## 2. Materials and Methods 

### 2.1. Materials

All reagents were commercially available and consumed as received. Nickel (II) nitrate hexahydrate (Ni(NO_3_)_2_; 6H_2_O ≥ 99%, Merck, Darmstadt, Germany), 2,5-dihyroxyterephtalic acid (DOT 98%, Aldrich), N,N-dimethylformamide (DMF 99.8%, Merck), ethanol absolute (99.8%, BDH), and methanol (MeOH, 99.9%, Merck) were all purchased from Portray (M) Sdn Bhd. Ultra-high pure CO_2_ (99.99%) was purchased from Linde Gas Malaysia Sdn Bhd (Petaling Jaya, Malaysia).

### 2.2. Synthesis of Ni-MOF-74

**Figure 1:**
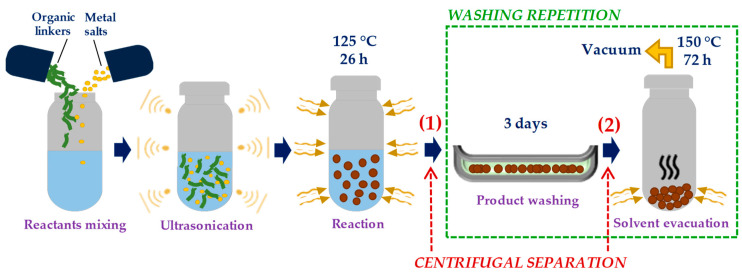
Schematic figure of solvothermal synthesis of this work with repeating washing processes or with additional centrifugal separations.

The following preparation is the typical procedure of solvothermal synthesis of MOF-74 materials reported elsewhere [20,21,22,23,24,25,26], noting that the synthesis parameters might differ. A 3.3:1 (mol/mol) ratio of Ni(NO_3_)_2_·6H_2_O and DOT linker was completely dissolved in a 15:1:1 (v/v/v) mixture of DMF, ethanol, and distilled water using ultrasonication. The homogeneous solution was then transferred into a 125 mL Teflon reactor (Kean Seng Engineering Works, Pusing, Malaysia) and stainless steel, and left in the oven at 125 °C for 26 h. The reaction product (MOF powders) was immersed in methanol for 3 days with 6-time replenishment of fresh methanol. The product was recovered, and, remaining solvent was evacuated under a dynamic vacuum at 150 °C for 72 h. The synthesis processes are summarized in Figure 1. The produced MOFs were denoted as the sample of the first-batch synthesis.

### 2.3. Optimization of Washing Processes 

For the first-batch synthesis, the washing processes were repeated to improve the separation of MOF product from the reactive medium, marked green in Figure 1. For the second-batch synthesis, the centrifugal separations were introduced in the synthesis routes (without any washing repetition) at two different points, marked red in Figure 1: (1) Between reaction and product washing, and (2) between product washing and solvent evacuation. Methanol was used as the washing solvent in centrifugation. A solution of 360 mg of MOF powder and fresh methanol was poured into six centrifuge tubes for 50 mL each. An optimization study of centrifugal time and rotation speed was carried out to obtain the best separation quality. It consisted of varying the parameters one by one and observing the separation between MOF products and methanol after each centrifugation. The incomplete separation was indicated by the dispersion of MOF particles in methanol shown by murky methanol color, indicating that both centrifugal parameters were insufficient. A good separation was observed by total sedimentation of MOFs, but without any detachment of MOF “flakes” from the sedimentation, failing which either centrifugal time or rotation speed was overestimated. As a result, the optimized centrifugal time and rotation speed for 50 mL solution of 60 mg MOFs in methanol were determined as 7 min and 8000 rpm, respectively. The produced MOFs were denoted as the sample of the second-batch synthesis.

### 2.4. Characterization of Materials 

The crystalline structure of the synthesized samples was analyzed using powder X-ray diffraction (PXRD). The patterns were recorded by using Bruker AXS-D8 Advance Diffractometer (Billerica, MA, USA) with Cu Kα radiation (λ = 1.5406 Å), step size of 0.02° in 2θ ranging from 5° to 45°, and scan rate of 1°/min. A novaculite was used as the standard material to determine instrumental resolution of the diffractometer. Fourier-transform infrared spectroscopy (FTIR) analysis via potassium bromide (KBr) method was used to generate spectra with patterns that provide structural insights by using Thermo Nicolet IS5 Spectrometer (Waltham, MA, USA) in wave number range of 4000–400 cm^−1^ with 32 number of scan. For every sample, the background scanning was performed to recognize the presence of air impurities such as carbon dioxide peak.

### 2.5. Adsorption Isotherm Measurements

CO_2_ adsorption isotherm measurements of the synthesized materials were obtained in a pressure range of 0–1.2 bar and at 25 °C using BELSORP-mini II from MicrotracBEL (Osaka, Japan). For the second adsorption cycles onwards, the sample was outgassed at the same condition as solvent evacuation prior to adsorption measurement. The adsorption performances were evaluated for Ni-MOF-74 of the first-batch synthesis without and with washing repetition, and also, for Ni-MOF-74 of the second-batch synthesis with centrifugation 1 and both centrifugations 1 and 2.

## 3. Results and Discussions

Ni-MOF-74 powder was successfully synthesized and confirmed by the PXRD result, which was in agreement with the diffractions calculated from the reference structure available in Cambridge Crystallographic Data Centre (CCDC, FIJDOS 265095). In order to assess the quality of the material produced from the first-batch synthesis, ensuring the efficiency of pore activation, CO_2_ adsorption isotherms in a pressure range of 0–1.2 bar and at 25 °C were measured. It is observed that the CO_2_ uptake of the first adsorption cycle is 0.34 mmol/g at 1 bar, which is far below the reported value of 5.08 mmol/g of the well-known work of Caskey et al. at the same operating conditions [20]. The sample was outgassed and the following adsorption/desorption cycles were performed resulting in the slight progressive increment in CO_2_ uptakes: 0.38 mmol/g (second) and 0.85 mmol/g (third). This observation figures that the solvent evacuation has been incomplete during the synthesis procedure because some micropores have been activated in the progressive outgassing upon multiple adsorption/desorption cycles. It means that the washing processes of MOFs in which the reactive medium, especially DMF “pore template”, which should have been removed, was not carried out properly. Then, the washing processes were repeated for the same sample followed by solvent evacuation. It is observed that the CO_2_ uptake has increased to 1.22 mmol/g, but the value is still further away from the expectation even after successive washing repetitions. It is understood that the technique of the washing processes needs improvement.

**Figure 2:**
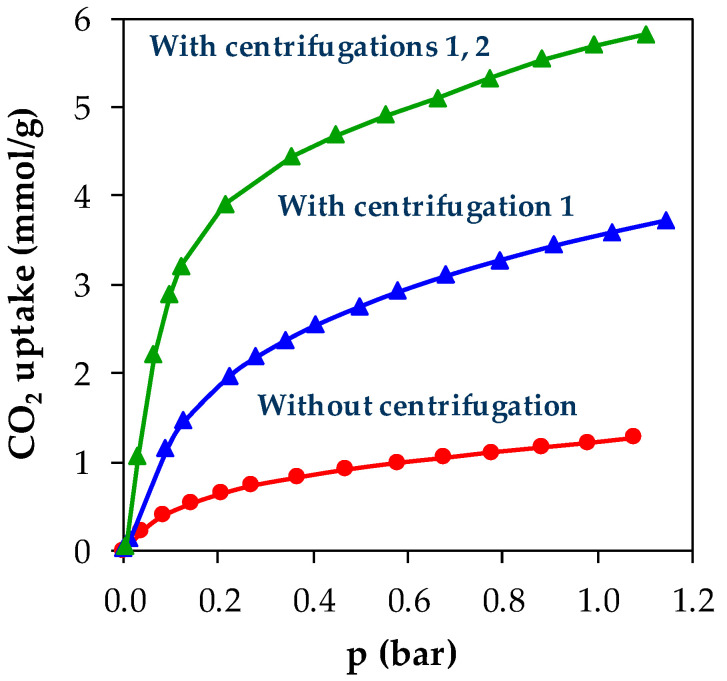
CO_2_ adsorption isotherms of Ni-MOF-74 samples from the first-batch synthesis after washing repetitions (red) and the second-batch synthesis with centrifugation 1 (blue) and centrifugations 1 and 2 (green), in a pressure range of 0–1.2 bar and at 25 °C.

For the second-batch synthesis, centrifugal separations were introduced at two different points as shown in Figure 1 with the aim of: (1) Removing the reactive medium from the MOF powder after the reaction, and, (2) separating the polluted methanol from the MOF powder after the product washing. Two Ni-MOF-74 samples were synthesized in the second-batch synthesis; one was prepared with the addition of centrifugation 1 and another one with centrifugations 1 and 2. The effect of centrifugal separations on the pore activation of the MOFs was then assessed by performing CO_2_ adsorption measurement in a pressure range of 0–1.2 bar and at 25 °C. The results were presented in Figure 2 comparing the isotherm of the first-batch sample with washing repetitions (red). At 1 bar, the second-batch samples display higher CO_2_ uptake with 3.70 mmol/g for centrifugation 1 (blue) and 5.80 mmol/g for centrifugations 1 and 2 (green). The final value is comparable to the above-mentioned literature data. It shows that the introduction of the centrifugal separation technique to the synthesis routes at two points has evidently improved the separation in the washing processes.

**Figure 3:**
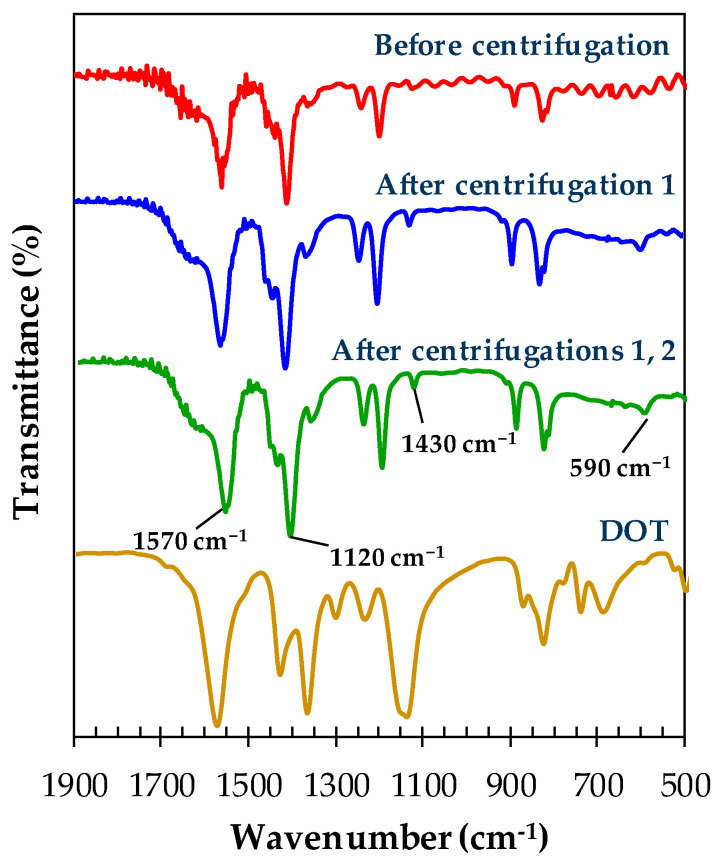
FTIR spectra of Ni-MOF-74 samples from before centrifugation (red), after centrifugation 1 (blue), and after centrifugations 1 and 2 (green), with the spectrum of 2,5-dihyroxyterephtalic acid linker (brown) from [30].

The characterization of materials was carried out using FTIR to analyze the organic chemical structure of the samples as a function of centrifugal separation as presented in Figure 3. The red, blue, and green spectra represent the reaction products before centrifugation, after centrifugation 1, and after centrifugations 1 and 2, respectively. All samples have the same characteristic peaks of symmetric (1570 cm^−1^) and asymmetric (1430 cm^−1^) stretching vibrations of carboxylate groups [27], which are the functional groups of DOT linkers that act as organic fragments in Ni-MOF-74. All samples also exhibit characteristic peaks in 1370–1000 cm^−1^ and 1000–500 cm^−1^ regions assigned for in-plane and out-of-plane C-H bending vibrations, respectively [27]. By applying the same resolution and condition of acquisition of the analysis, it is noticed that the green and blue spectra produce peaks with higher intensities whereby the intensity in FTIR spectroscopy is directly proportional to the concentration of molecules of interest in a sample [28]. The equation that relates concentration to absorbance is known as Beer’s law which is presented below [29]:

(1) A=εlc

where A is absorbance, ε is absorptivity, l is path length, and c is concentration. Thus, it indicates that the concentrations of molecules of MOFs in the sample are significantly improved after centrifugal separation. The reason behind this is probably due to the loss of impurities (residual reactants and remaining solvents) that had been separated by centrifugal separation, leading to higher purity or concentration of MOFs in the sample. The feeble intensities in the red spectrum give rise to the oscillating small peaks in C-H bending regions, so that the two characteristic peaks of Ni-MOF-74 at 1120 cm^−1^ (in-plane bending) and 590 cm^−1^ (out-of-plane bending) are not visible. Despite exhibiting different values of adsorption capacity, it can be seen that there was no significant difference in FTIR spectra between the second-batch samples after centrifugation 1 and after centrifugation 1 and 2, indicating that the impurities have been largely removed from both samples. The impurities that were present after centrifugation 1 were considerably negligible to the overall concentration of MOFs, leading to the similar FTIR spectra with the sample after centrifugation 1 and 2. On the other hand, it can be understood that the step of classical product washing (immersion in methanol) was not able to “wash” the contaminated MOF products as it was the case for the first-batch sample.

**Figure 4:**
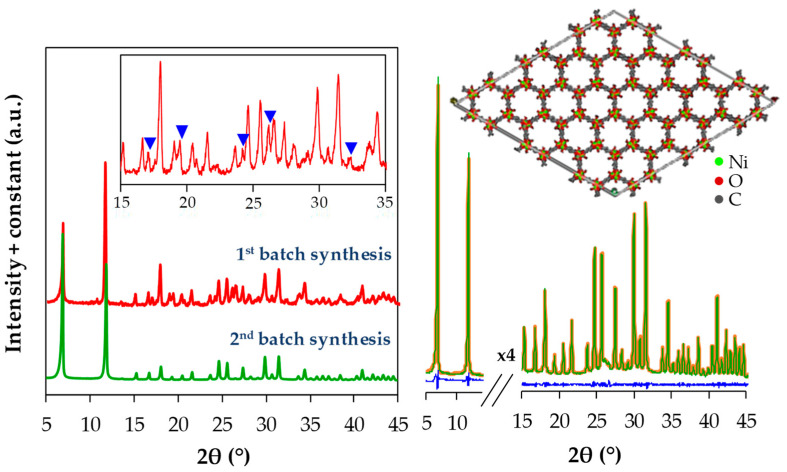
(left) Powder X-ray diffraction (PXRD) patterns of Ni-MOF-74 samples from the first-batch synthesis (red) and the second-batch synthesis with centrifugations 1 and 2 (green). (right) Results of Rietveld refinement on the second-batch sample; green, yellow, and blue lines represent experimental data, indexed reflections of molecular structure of Ni-MOF-74, and difference plot, respectively.

PXRD analysis was performed to analyze the crystalline structure of Ni-MOF-74 samples prepared before and after the optimization of the synthesis routes. As presented in Figure 4, in general, the samples from the first-batch synthesis and the second-batch synthesis with centrifugation 1 and 2, produce similar reflections characteristic of Ni-MOF-74. However, it is observed that the level of background noises indicating the amount of amorphous phases in the sample have been significantly decreased after centrifugations, indicating the enhancement in crystallinity of the frameworks [31,32,33,34,35,36]. The relative amorphicity in the first-batch sample may be attributed to the presence of residual reactants, especially soluble salts and DOT that consist of similar diffraction atoms with those in the frameworks. It was reported in various fields of application of MOFs that the intensity of XRD reflections of MOFs charged with organic compounds decreased in comparison to the bare MOFs due to the increment in amorphicity [31,32,33,34,35,36]. Besides that, the presence of some reflections in the first-batch samples, which do not belong to Ni-MOF-74 (marked with blue triangles), signify the interference of impurities. This observation is in accordance with FTIR analysis as well as CO_2_ adsorption measurements whereby the centrifugations have improved the separation leading to the effective pore activation and thus satisfy gas adsorption uptake. In order to assess the crystallographic data of the final sample from the second-batch synthesis, Rietveld refinement of the unit cell was carried out using PANalytical X’Pert HighScore Plus software, which is done by the least squares fit method through the angular differences between measured reflections and indexed reflections [37]. The results reveal the best fit simulation affording a nearly perfect zero difference plot with an acceptable value of fitting goodness. As presented in Table 1, the final sample exhibits comparable values of lattice parameters of the unit cell to the reference structure, which are given in Angstrom (Å) and considered accurate to ±3 (for *a* and *b*) and ±1 (for *c*) in the last reported decimal.

Table 2 recapitulates the stages of optimization of the washing processes. The comparison results of CO_2_ adsorption uptake of this work to the several literature data of Ni-MOF-74 at 1 bar and 25 °C are summarized in Table 3. It can be seen that the synthesized material of this work exhibits the encouraging result and is competitive to the existing published data.

## 4. Conclusions

In order to have proper activation of MOF pores, the washing processes in solvothermal synthesis of Ni-MOF-74 were optimized. Centrifugal separations were introduced in the synthesis routes first, to remove the reactive medium from the product after the reaction, and second, to separate the polluted methanol from the product after the washing. The sample produced from the optimized synthesis routes displayed significantly higher CO_2_ uptake in comparison to the sample of the basic synthesis. The CO_2_ adsorption uptake gained by the final sample was 5.80 mmol/g at 1 bar and 25 °C, which was competitive with the published data. The outcome indicated the effectiveness of the separation in the washing processes leading to the complete activation of MOF pores.

## Figures and Tables

**Table 1:** Crystallographic data of synthesized Ni-MOF-74.

Ni-MOF-74 Sample	Second-Batch Synthesis	Calculated from CCDC, FIJDOS 265095
Crystal system	Trigonal (hexagonal axes)	
Angles	α = β = 90°, γ = 120°	
Space group	R-3	
Lattice parameters		
*a* = *b* (Å)	26.009 (±3)	26.026
*c* (Å)	6.777 (±1)	6.759
Unit cell volume (Å^3^)	3970	3965
Rexp	2.39	
Rp	3.32	
Rwp	4.67	
Goodness of fit	1.95	

**Table 2:** Stages of optimization of washing processes in solvothermal synthesis.

Stage	Synthesis	CO_2_ Adsorption Uptake	Observation
1	First batch	Increases from 0.34 to 0.85 mmol/g after three successive adsorption cycles	Activation of some micropores by progressive outgassing.
2	First batch with washing repetitions	1.22 mmol/g	Activation of some micropores by more times of washing.
3	Second batch with centrifugation (1)	3.70 mmol/g	Good separation of reactive medium from MOF powder.
4	Second batch with centrifugations (1) and (2)	5.80 mmol/g	Good separation of reactive medium and washing solvents from MOF powder.

**Table 3:** Comparison results of CO_2_ adsorption uptake of Ni-MOF-74 at 1 bar and 25 °C with literature data.

Reference	This Work	[20]	[26]	[15]	[25]
CO_2_ Uptake (mmol/g)	5.80	5.08	4.00	3.75	2.50

## References

[1] Eddaoudi M., Kim J., Rosi N., Vodak D., Wachter J., O’keeffe M., Yaghi O.M. (1999). Systematic design of pore size and functionality in isoreticular MOFs and their application in methane storage. Science.

[2] Banerjee D., Wang H., Deibert B.J., Li J., Kaskel S. (2016). Chapter 4: Alkaline earth metal-based metal-organic frameworks: Synthesis, Properties and Applications. The Chemistry of Metal-Organic Frameworks: Synthesis, Characterization, and Applications.

[3] Yusran Y., Xu D., Fang Q., Zhang D., Qiu S. (2017). MOF-derived Co@NC nanocatalyst for catalytic reduction of 4-nitrophenol to 4-aminophenol. Micropor. Mesopor. Mater..

[4] Pentyala V., Davydovskaya P., Pohle R., Urban G., Yurchenko O. (2014). Mg-MOF74 and Co-MOF74 as sensing layers for CO_2_ detection. Procedia Eng..

[5] Horcajada P., Serre C., Vallet-Regí M., Sebban M., Taulelle F., Férey G. (2006). Metal-organic frameworks as efficient materials for drug delivery. Angew. Chem. Int. Ed..

[6] Khan N.A., Hasan Z., Jhung S.H. (2013). Adsorptive removal of hazardous materials using metal-organic frameworks (MOFs): A review. J. Hazard. Mater..

[7] Ahmed E., Deep A., Kwon E.E., Brown R.J.C., Kim K.-H. (2016). Performance comparison of MOF and other sorbent materials in removing key odorants emitted from pigpen slurry. Sci. Rep..

[8] Liu B., Shioyama H., Jiang H., Zhang X., Xu Q. (2010). Metal-organic framework (MOF) as a template for syntheses of nanoporous carbons as electrode materials for supercapacitor. Carbon.

[9] Cui Y., Yue Y., Qian G., Chen B. (2011). Luminescent functional metal-organic frameworks. Chem. Rev..

[10] Dey C., Kundu T., Biswal B.P., Mallick A., Banerjee R. (2014). Crystalline metal-organic frameworks (MOFs): Synthesis, structure and function. Acta Crystallogr. Sect. B.

[11] Lopez M., Canepa P., Thonhauser T. (2013). NMR study of small molecule adsorption in MOF-74-Mg. J. Chem. Phys..

[12] Valenzano L., Civalleri B., Chavan S., Palomino G.T., Areán C.O., Bordiga S. (2010). Computational and experimental studies on the adsorption of CO, N_2_, and CO_2_ on Mg-MOF-74. J. Phys. Chem. C.

[13] Yang D.-A., Cho H.-Y., Kim J., Yang S.-T., Ahn W.-S. (2012). CO_2_ capture and conversion using Mg-MOF-74 prepared by a sonochemical method. Energy Environ. Sci..

[14] Cho H.-Y., Yang D.-A., Kim J., Jeong S.-Y., Ahn W.-S. (2012). CO_2_ adsorption and catalytic application of Co-MOF-74 synthesized by microwave heating. Catal. Today.

[15] Wu X., Bao Z., Yuan B., Wang J., Sun Y., Luo H., Deng S. (2013). Microwave synthesis and characterization of MOF-74 (M = Ni, Mg) for gas separation. Micropor. Mesopor. Mater..

[16] Chen Y.W., Lv D.F., Wu J.L., Xiao J., Xi H.X., Xia Q.B., Li Z. (2017). A new MOF-505@GO composite with high selectivity for CO_2_/CH_4_ and CO_2_/N_2_ separation. Chem. Eng. J..

[17] Lee Y.-R., Kim J., Ahn W.-S. (2013). Synthesis of metal-organic frameworks: A mini review. Korean J. Chem. Eng..

[18] Yi T.-F., Jiang L.-J., Shu J., Yue C.-B., Zhu R.-S., Qiao H.-B. (2010). Recent development and application of Li_4_Ti_5_O_12_ as anode material of lithium ion battery. J. Phys. Chem. Solids.

[19] Larabi C. (2011). Surface Organometallic Chemistry on Metal Organic Frameworks (MOF): Synthesis, Characterization and Their Application in Catalysis. Ph.D. Thesis.

[20] Caskey S.R., Wong-Foy A.G., Matzger A.J. (2008). Dramatic tuning of carbon dioxide uptake via metal substitution in a coordination polymer with cylindrical pores. J. Am. Chem. Soc..

[21] Britt D., Furukawa H., Wang B., Glover T.G., Yaghi O.M. (2009). Highly efficient separation of carbon dioxide by a metal-organic framework replete with open metal sites. Proc. Natl. Acad. Sci. USA.

[22] Dietzel P.D.C., Besikiotis V., Blom R. (2009). Application of metal-organic frameworks with coordinatively unsaturated metal sites in storage and separation of methane and carbon dioxide. J. Mater. Chem..

[23] Bao Z., Yu L., Ren Q., Lu X., Deng S. (2011). Adsorption of CO_2_ and CH_4_ on a magnesium-based metal organic framework. J. Colloid Interface Sci..

[24] Lou W., Yang J., Li L., Li J. (2014). Adsorption and separation of CO_2_ on Fe(II)-MOF-74: Effect of the open metal coordination site. J. Solid State Chem..

[25] Chen D.-L., Shang H., Zhu W., Krishna R. (2014). Transient breakthroughs of CO_2_/CH_4_ and C_3_H_6_/C_3_H_8_ mixtures in fixed beds packed with Ni-MOF-74. Chem. Eng. Sci..

[26] Adhikari A.K., Lin K.-S. (2016). Improving CO_2_ adsorption capacities and CO_2_/N_2_ separation efficiencies of MOF-74 (Ni, Co) by doping palladium-containing activated carbon. Chem. Eng. J..

[27] Stuart B.H. (2004). Chapter 4: Organic molecules. Infrared spectroscopy: Fundamentals and applications.

[28] Griffiths P.R. (2006). Beer’s Law. Handbook of Vibrational Spectroscopy.

[29] Maikala R.V. (2010). Modified Beer’s law-historical perspectives and relevance in near-infrared monitoring of optical properties of human tissue. Int. J. Ind. Ergon..

[30] Kamal K., Grekov D., Hamon L., Shariff A.M., Bustam M.A., Pré P. (2020). Improving textural properties of magnesium-based metal-organic framework for gas adsorption by doping carbonaceous material. Micropor. Mesopor. Mater..

[31] Haydar M.A.L., Abid H.R., Sunderland B., Wang S. (2017). Metal organic frameworks as a drug delivery system for flurbiprofen. Drug Des. Dev. Ther..

[32] Dutta R., Rao M.N., Kumar A. (2019). Investigation of ionic liquid interaction with ZnBDC-metal organic framework through Scanning eXAfS and inelastic neutron Scattering. Sci. Rep..

[33] Sun X., Xia Q., Zhao Z., Li Y., Li Z. (2014). Synthesis and adsorption performance of MIL-101(Cr)/graphite oxide composites with high capacities of n-hexane. Chem. Eng. J..

[34] Huang W., Zhou X., Xia Q., Peng J., Wang H., Li Z. (2014). Preparation and adsorption performance of GrO@Cu-BTC for separation of CO_2_/CH_4_. Ind. Eng. Chem. Res..

[35] Zhao Y., Cao Y., Zhong Q. (2014). CO_2_ capture on metal-organic framework and graphene oxide composite using a high-pressure static adsorption apparatus. J. Clean Energy Technol..

[36] Yu Z., Deschamps J., Hamon L., Prabhakaran P.K., Pré P. (2017). Hydrogen adsorption and kinetics in MIL-101 (Cr) and hybrid activated carbon-MIL-101 (Cr) materials. Int. J. Hydrog. Energy.

[37] Speakman S.A. (2011). Fundamentals of Rietveld Refinement, II. Refinement of a Single Phase. MIT Cent. Mater. Sci. Eng..

